# Occurrence, Source Apportionment, and Risk Assessment of Antibiotics in Mangrove Sediments from the Lianzhou Bay, China

**DOI:** 10.3390/antibiotics13090820

**Published:** 2024-08-28

**Authors:** Pengfei Sun, Yongyu Tan, Zuhao Zhu, Tinglong Yang, Shalini Thevarajan, Li Zhang

**Affiliations:** 1 Guangxi Beibu Gulf Key Laboratory of Marine Resources, Environment and Sustainable Development, Fourth Institute of Oceanography, Ministry of Natural Resources, Beihai 536000, China; sunpengfei@4io.org.cn (P.S.); tanyongyu@4io.org.cn (Y.T.); zhuzuhao@4io.org.cn (Z.Z.); yangtinglong@4io.org.cn (T.Y.); shalini@zju.edu.cn (S.T.); 2 Key Laboratory of Tropical Marine Ecosystem and Bioresource, Fourth Institute of Oceanography, Ministry of Natural Resources, Beihai 536000, China; 3 School of Environmental Science and Engineering, Nanjing University of Information Science and Technology, Nanjing 210044, China; 4 Ocean College, Zhejiang University, Zhoushan 316021, China

**Keywords:** antibiotic, mangrove sediment, source analysis, distribution, risk assessment

## Abstract

In recent years, the widespread application of antibiotics has raised global concerns, posing a severe threat to ecological health. In this study, the occurrence, source, and ecological risks of 39 antibiotics belonging to 5 classes in mangrove sediments from Lianzhou Bay, China, were assessed. The total concentrations of the antibiotics (∑39 antibiotics) ranged from 65.45 to 202.24 ng/g dry weight (dw), with an average of 142.73 ± 36.76 ng/g dw. The concentrations of these five classes of antibiotics were as follows: Sulfonamides (SAs) > Tetracyclines (TCs) > Fluoroquinolones (QUs) > Penicillin (PCs) > Macrolides (MLs). The spatial distribution of antibiotics varied as high tidal zone > middle tidal zone > low tidal zone. The total organic carbon (TOC), pH, nitrate (NO_3_^−^-N), and nitrite (NO_2_^−^-N) of the sediment significantly influenced the distribution of antibiotics (*p* < 0.05). A source analysis identified untreated sewage from aquaculture as the primary source of antibiotics in the local mangrove. A risk assessment revealed that ciprofloxacin, norfloxacin, ofloxacin of QUs, and tetracycline of TCs exhibited medium risks to algae in certain sampling sites, while other antibiotics exhibited low or no risks to all organisms. Nevertheless, the total risk of all the detected antibiotics to algae was medium in 95% of the sites. The overall ecological risk level of antibiotics in the middle tidal zone was slightly lower than in the high tidal zone and the lowest in the low tidal zone. In summary, the experimental results provided insights into the fate and transport behaviors of antibiotics in mangrove sediments from Lianzhou Bay.

## 1. Introduction

Antibiotics, classified as antibacterial and bactericidal drugs, are commonly used to treat or prevent bacterial infections in humans and animals [1]. Over the years 2000 to 2015, global antibiotic consumption has increased by 65% from 21.1 to 34.8 billion defined daily doses (DDDs) [2]. In 2013, China utilized a total of 36 commonly used antibiotics, accounting for 92,700 tons, with 48% used for human health and the rest for animals [3]. Predictions indicate that the consumption of veterinary antibiotics in China will double by 2030 [2]. Antibiotics have been widely detected in various environments, including sewage treatment plants, aquatic ecosystems, and livestock waste due to their widespread use, incomplete breakdown, and insufficient wastewater treatment [4,5,6]. In coastal areas, antibiotics can have long-term effects on aquatic organisms [1]. The toxic effects of antibiotics on green algae are mainly attributed to the inhibition of chloroplast metabolisms, such as protein synthesis and photosynthesis, which affect cell growth [7]. The effects of antibiotics on ecological functions can lead to changes in nitrogen transformation, methanogenesis, sulfate reduction, nutrient cycling, and organic matter degradation [8]. Moreover, the excessive use of antibiotics can increase the abundance of antibiotic resistance genes (ARGs) among bacteria, especially harmful pathogens threatening human health [9]. Antibiotics are increasingly recognized as an emerging environmental pollutant [10]. Therefore, understanding the environmental impacts associated with antibiotic use is crucial.

Mangroves, composed of highly coordinated mangrove plants, animals, and microorganisms, are aquatic ecosystems distributed in tropical and subtropical areas [11]. As a type of intertidal zone [12], mangrove wetlands are renowned for their high biological productivity and abundant total organic carbon (TOC) [13], facilitating pollutant decomposition and nutrient cycling by effectively processing and utilizing terrestrial anthropogenic emissions before they reach the ocean [14]. However, mangroves face multiple threats, including sea-level rise [15], deforestation [16], hyper-salination, and contamination by wastewater containing trace metals, various persistent organic pollutants [17], and pharmaceuticals such as antibiotics [18,19,20]. These pollutants significantly impact the microbial diversity of mangroves [21], which play crucial roles in ecosystem productivity [22], nutrient cycling [23], and the synthesis of various metabolites such as antimicrobial compounds [24]. A study by Liu et al. [11] on the coastal mangroves in southern China revealed that the total antibiotic concentrations were higher (>250 ng/g dw) in the mangrove sediment of Fangchenggang (mean, 501 ng/g dw), Hong Kong (mean, 368 ng/g dw), Zhanjiang (mean, 311 ng/g dw), and Shenzhen (mean, 268 ng/g dw). Therefore, mangrove sediments are considered suitable indicators of past human activity in the study area [25]. However, reports on the occurrence and distribution of antibiotics in the mangrove wetlands area are scarce. Furthermore, the source and potential ecological risks posed by these antibiotics to the surrounding ecosystems remain largely unexplored.

With the rapid development of the Beibu Gulf Economic Rim, the increased industrialization and population have significantly impacted the environmental quality of this region [25,26,27]. The advantageous geographical location renders the Beibu Gulf an ideal area for the aquaculture industry. In 2017, the total farmed seafood production in this region was 1.3 million tons [28], inevitably leading to the contamination of antibiotics. Lianzhou Bay is located on the northern margin of Beibu Gulf and is mostly characterized as a faulted estuarine bay, receiving inflow from the Nanliu River, Fengfeng River, Lianzhou River, and Qixing River, collectively forming an estuarine delta [29]. The tide in Lianzhou Bay is moderate, with a flow rate of high tide being lower than that of low tide. Consequently, sediment, debris, and other materials carried by the sea tide and river inflows are deposited on the beach. These soil conditions are conducive to the growth of mangroves, thereby establishing Lianzhou Bay as one of the principal bays for mangrove distribution in Guangxi [30]. Tidal patterns play a vital role in the spatial distribution of antibiotics [31]. Zhang et al. [32] showed that antibiotic concentrations in the East River estuary were influenced by tidal fluctuations. The moderate tidal velocity in Lianzhou Bay may facilitate the deposition of antibiotics in mangrove sediments, posing ecological risks. Therefore, this study has selected mangrove wetlands in Lianzhou Bay as a representative area and 39 commonly used antibotics belonging to 5 different classes. It aims to (1) analyze the occurrence and spatial distribution of antibiotics in mangrove sediments collected from the high, middle, and low tidal zones in Lianzhou Bay, China; (2) determine the main factors influencing the distribution of target antibiotics in the sediments and explore the potential sources, and (3) evaluate the ecologic risks of detected antibiotic residues in mangrove sediments to various organisms. 

## 2. Results and Discussion

### 2.1. Occurrence and Distribution of Antibiotics in Mangrove Sediments from Lianzhou Bay

In this study, 28 out of 39 target antibiotics were detected in mangrove sediments. The detection frequencies of Norfloxacin (NOR), Ciprofloxacin (CIP), Enrofloxacin (ERX), Azithromycin (AZM), Roxithromycin (ROX), Ofloxacin (OFX), Erythromycin (ERY), Sulfapyridine (SPD), Sulfadiazine (SA), Sulfamethoxazole (SMX), Sulfamerazine (SMZ), Sulfamethoxine (SMM), Oxytetracycline (OXY), Chlortetracyelin (CTE), Tetracycline (TC), and Doxycycline (DOX) were >90%, revealing the widespread existence of target antibiotics in the mangrove sediments of Lianzhou Bay. The total antibiotic concentrations (∑_39_ antibiotics) at 21 sites ranged from 65.45 to 202.24 ng/g dry weight (dw), with an average value of 142.73 ± 36.76 ng/g dw (Table S1). The mean antibiotic concentration in this study was lower than that observed in mangrove sediments from Fangcheng Bay (mean, 501 ng/g dw), Hong Kong (mean, 368 ng/g dw), Zhanjiang province (mean, 311 ng/g dw), and Shenzhen (mean, 268 ng/g dw) [11]. On average, the concentration of the SAs (15.25–100.07 ng/g dw), TCs (20.74–65.33 ng/g dw), QUs (18.53–51.10 ng/g dw), PCs (0.00–7.40 ng/g dw), and MLs (0.58–2.89 ng/g dw) accounted for 46%, 27%, 25%, 0.9%, and 0.6% of the total antibiotics, respectively. The SAs, TCs, and QUs were the main detected antibiotics, which was consistent with those reported in the sediments from the Gaoqiao mangroves in Zhanjiang, China [33,34]. SAs are frequently used in human medicine, livestock agriculture, and aquaculture, as well as growth enhancers [35]. Qiu et al. [36] found high concentrations of SAs in the sediments of the Shenzhen River estuary. Zhang et al. [37] observed that high concentrations of SAs could hinder microbial activity, thus retarding the degradation of SAs in sediments. TCs are the second most widely used antibiotics due to their advantages of low cost and broad spectrum and high antimicrobial activity [38]. Currently, TCs have been detected in various water environments and sediments [9,11]. QUs were the third most abundant antibiotics, with mean values of 36.29 ± 9.59 ng/g dw, which was much lower than that in coastal mangrove sediments of Fangchenggang (mean, 227.7 ng/g dw), Zhanjiang (mean, 108.8 ng/g dw), Shenzhen (mean, 119.2 ng/g dw), Hong Kong (mean, 126.6 ng/g dw), and Yunxiao (mean, 93.0 ng/g dw) in China [11]. Studies have indicated that QUs have a high chelating capacity towards cations and can bind with particulate matter, making them easily adsorbed in the sediments [39,40].

**Figure 1:**
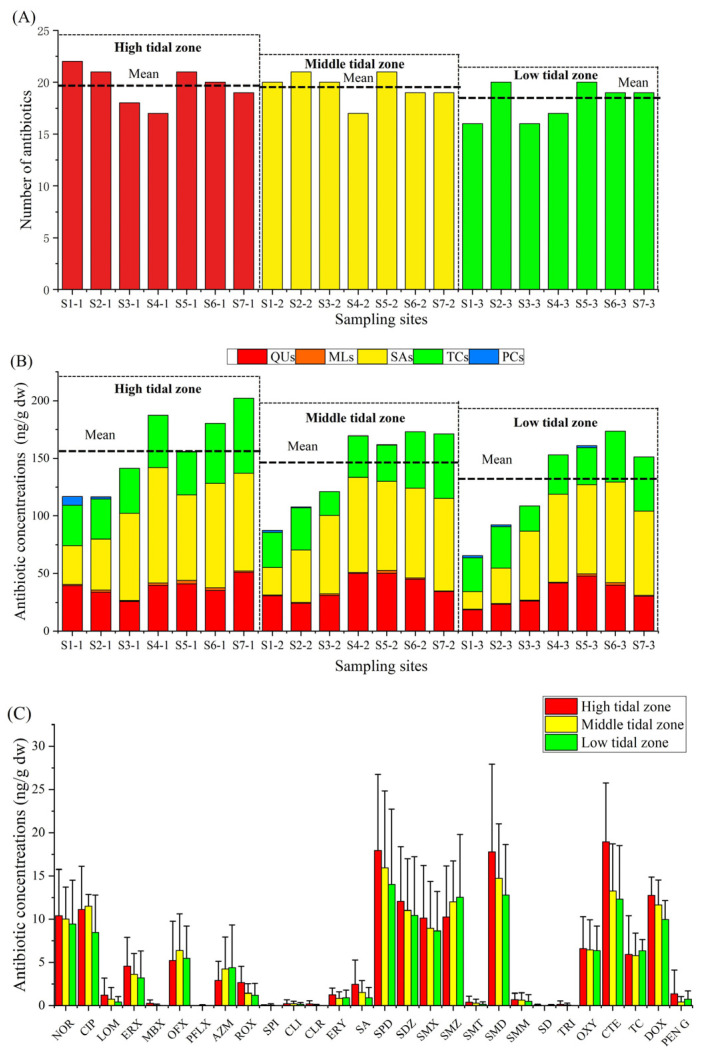
Presence (**A**), concentration composition (**B**), and regional distribution (**C**) of antibiotics in mangrove sediments.

The numbers of detected antibiotics in high, middle, and low tidal zones were 25, 25, and 22, respectively (Figure 1A). There was no significant difference between the number of antibiotics among the three tidal zones. The mean antibiotic concentration in the high tidal zone was the highest (157.54 ± 33.84 ng/g dw), followed by that in the middle tidal zone (141.77 ± 35.39 ng/g dw), and the low tidal zone (129.36 ± 40.52 ng/g dw) (Figure 1B). Nevertheless, there were no significant differences between the mean antibiotic concentration among the three tidal zones. The difference in the distribution of antibiotics in the mangroves of Lianzhou Bay may be due to the direct discharge of wastewater from the local aquaculture pond with high antibiotic concentrations into the mangrove environment [11]. Additionally, the mangrove ecosystem has the capacity to accumulate or degrade various pollutants (antibiotics, trace metals, polycyclic aromatic hydrocarbons, etc.) from the land area [19,34]. Therefore, the mangrove ecosystem close to sewage outlets plays a crucial role in degrading significant amounts of antibiotics in sediments.

The mean concentration of each antibiotic distributed in each tidal zone is shown in Figure 1C. Chemicals with a low concentration (>5%) were not included in the following analysis of this paragraph. SMM, SDZ, SMX, SMD, NOR, CTE, and DOX was high tidal zone > middle tidal zone > low tidal zone. These antibiotics are frequently used in human medicine, aquaculture, and animal husbandry [35,41,42], and were preferentially discharged to the high tidal zone through sewage outflows, resulting in this distribution trend. The mean concentration of SMZ and TC in the low tidal zone were the highest among the three tidal zones. Han et al. [43] found that the main source of SMZ in the seawater of Laizhou Bay, Bohai Sea, was surrounding marine aquaculture. Another study had shown that SMZ in the seawater of the Beibu Gulf coastal area mainly originates from river discharges from livestock and poultry farming upstream [44]. Thus, tidal action and river inputs may lead to a large accumulation of SMZ carried by seawater and rivers in low-tide sediments. Some researchers have indicated that the TC concentration of the river water in the wet season was higher than that in the dry season [45]. As the survey time of this study was the wet season, the flowing of river water carried with a higher TC content may lead to TC accumulation in sediments of the low tidal zone. Moreover, the mean concentration of CIP and OFX in the middle tidal zone were the highest among the three tidal zones. Furthermore, the TOC and sediment particle size in the middle tidal zone were also the highest. Some studies have proved that a higher TOC and larger sediment particle size contribute to the significant accumulation of antibiotics [33,34,39,46], which may lead to a higher content of CIP and OFX in the middle tidal zone. In addition, it has been reported that antibiotics with log*K*_ow_ of less than 1 cannot be easily adsorbed by the root epidermis or actively pass through the plant cell membrane due to their strong hydrophilic effects [47,48,49]. Herein, the logK_ow_ values of the CIP and OFX were both less than 1, indicating that they were not easily absorbed and cleansed by the mangrove plants and were stored in the sediments.

### 2.2. Correlation between Environmental Factors and Antibiotic Concentrations

Several environmental factors, such as TOC, particle size, pH, and nutrient composition, have been identified as influential factors affecting the distribution and persistence of antibiotics [46,50,51]. In this study, the physicochemical properties of mangrove sediments were analyzed and are shown in Table 1. The pH of the high tidal zone was significantly lower than that of the middle and low tidal zones (*p* < 0.05), while the pH of the low tidal zone was the highest. The order of NO_3_−-N concentration was as follows: high tidal zone > middle tidal zone > low tidal zone, and there were significant differences between every two tidal zones (*p* < 0.05). The TOC concentration was the highest in the high tidal zone, followed by the middle and low tidal zones. Additionally, the NO_2_−-N, NH_4_^+^-N, and sediment particle size were the highest in the middle tidal zone, followed by the low and high tidal zones.

**Figure 2:**
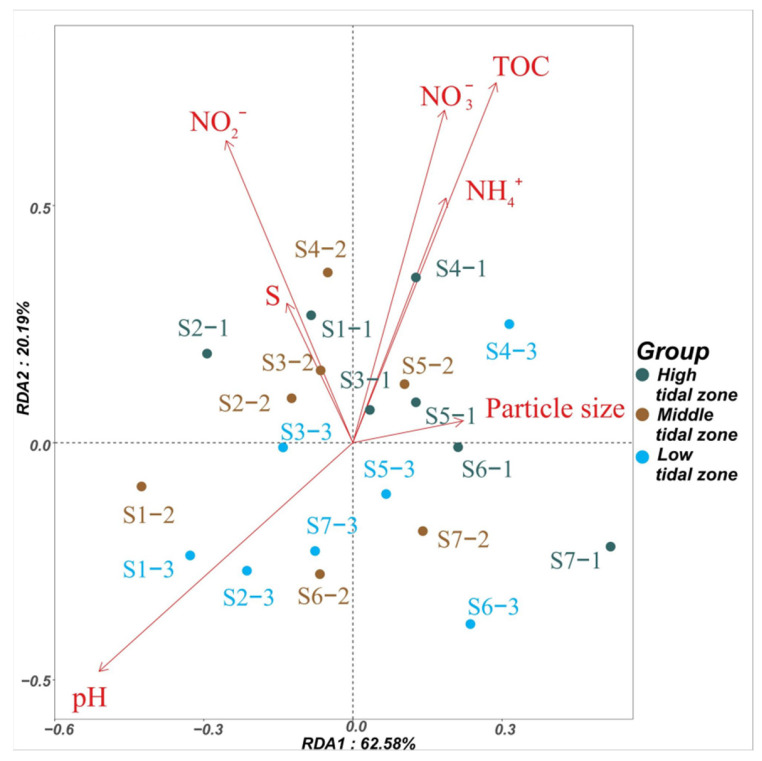
Redundancy analysis of the antibiotic concentrations and environmental factors in the sediment samples. The RDA1 and RDA2 explained 63% and 20% of the total variance, respectively.

To explore the correlation between the antibiotic concentrations and environmental factors of mangrove sediments, a Pearson analysis was conducted and the results are shown in Figure 2. The TOC, NO_3_−-N, NO_2_−-N, and pH accounted for 39% (*p* = 0.01), 32% (*p* = 0.03), 35% (*p* = 0.01), and 30% (*p* = 0.04) of the factors, revealing that the concentration distribution of antibiotics was strongly affected by these four environmental factors. TOC is a key environmental factor affecting antibiotic concentrations in sediments, and a higher TOC content can facilitate antibiotic absorption, leading to increased accumulation of antibiotics [34,39]. Previous studies have indicated that some antibiotics (SMX, TC, NOR, and CIP) can reduce the denitrification and anammox reactions by eliminating the denitrifying bacteria and decreasing their abundance, resulting in an increase in NO_3_−-N and NO_2_−-N levels [52,53,54]. Therefore, higher concentrations of NO_3_−-N and NO_2_−-N are associated with higher antibiotic concentrations in the antibiotic polluted areas [50]. Similarly, pH can affect the adsorption and biodegradability of antibiotics in sediments [55,56,57,58]. Herein, the antibiotic concentrations were significantly positively correlated (*p* < 0.01) with the TOC, NO_3_−-N, and NO_2_−-N in the high and middle tidal zones. Additionally, pH was significantly correlated with the antibiotic concentrations in the middle tidal zone (*p* < 0.05). Li et al. [59] also found a significant positive correlation (*p* < 0.05) between TOC and antibiotic concentrations in the sediments of the Pearl River estuary, China. Similarly, Chen et al. [60] found a significant positive correlation (*p* < 0.05) between NO_3_−-N and antibiotic concentrations in the sediments of Hailing Bay, China. The antibiotic concentrations in the low-tide zone were significantly positively correlated with the pH (*p* < 0.01). A previous study has reported a significant positive correlation between the pH and antibiotic concentrations (*p* < 0.05) in the sediments of typical bays of the East China Sea [51]. 

### 2.3. Potential Sources of Antibiotics in the Sediment of Lianzhou Bay

The sources of antibiotics in the sediment samples were identified using the varimax-rotated component matrix following PCA (Table 2). Chemicals with a low detection frequency (<10%) were not included in this study. Five principal components (PC1, PC2, PC3, PC4, and PC5), accounting for 25%, 17%, 17%, 15%, and 9% of the total variance, respectively, were identified. PC1 was highly associated with CIP, LOM, OFX, ROX, SPD, SMX, OXY, CTE, and TC. Studies have found that TCs, QUs, and SAs are the most frequently utilized antibiotics in aquaculture, with QUs displaying a significant and positive correlation with factors related to human healthcare (*p* < 0.05) [61]. CIP is also a commonly used antibiotic in aquaculture and has been detected in rivers in Italy and in major rivers in China [6,11,62]. TC is a commonly used antibiotic as a growth promoter in aquaculture to improve nutrient absorption capacity and enhance the body weight of aquatic organisms [63]. The presence of SMX and TC in mangrove sediments is probably due to the difficulty of their removal by conventional wastewater treatment plants [64,65]. Meanwhile, most of these antibiotic concentrations were significantly correlated with each other at the 0.01 level (Table S2), indicating that they may come from the same source or have the same environmental behaviors [66,67]. Thus, PC1 suggested that the source of antibiotics in the study area was the combined discharge of aquaculture and hospital wastewater. PC2 was highly associated with AZM, ERY, SA, SMZ, and SMM. Studies have reported that ERY is difficult to remove through conventional sewage treatment, with a removal rate below 20% [68,69]. Additionally, SMM is significantly positively correlated with the aquaculture (*p* < 0.05) [61]. Thus, PC2 suggested that the source of antibiotics in the study area was probably the combined sewage of human domestic and aquaculture. PC3 was highly associated with SDZ, SMZ, and SPD. PC3 suggested that the source of the SAs was aquaculture industry emissions [66]. PC4 was highly associated with ERX, AZM, SDZ, and SMD, and PC5 was highly associated with CTE and DOX. The detected antibiotics are commonly used in aquaculture, indicating that aquaculture activities are the main sources of the studied antibiotics in the mangrove sediments of Lianzhou Bay.

### 2.4. Risk Assessment 

With the widespread use of antibiotics, risk assessments of antibiotics in the environment have attracted considerable attention [4,70,71]. In this study, the ecological risks of the detected antibiotics to aquatic organisms at three trophic levels (including algae, fish, and invertebrates) were evaluated (Table S3). Without considering the combined toxicological contamination of each antibiotic, the RQ values of the antibiotics against the three categories of aquatic organisms (algae, fish, and invertebrate) were separately summed to obtain the RQ_sum_-algae, RQ_sum_-invertebrates, and RQ_sum_-fish. The RQ_sum_-algae was significantly higher than the RQ_sum_-invertebrate (*p* < 0.01) and RQ_sum_-fish (*p* < 0.01). This result implied that the algae was the most sensitive species to the detected antibiotics, and these antibiotics were not likely to pose risks to invertebrates and fish due to their low RQ_sum_ values. Similar results were observed in the sediments of the Beibu Gulf of China [1], Baiyangdian Lake of China [72], and the Hong Kong River of China [73]. Algae are important flora in mangrove ecosystems. As the high plant productivity in mangroves is only possible due to interactions with microorganisms, for example, cyanobacteria may contribute to these ecosystems by providing fixed nitrogen, carbon, and plant defense molecules, biosorption and bioremediation of xenobiotics, and secretion of substances that promote plant growth [74]. Thus, the high ecological risk of antibiotics to algae affects the ecological stability of mangroves.

**Figure 3:**
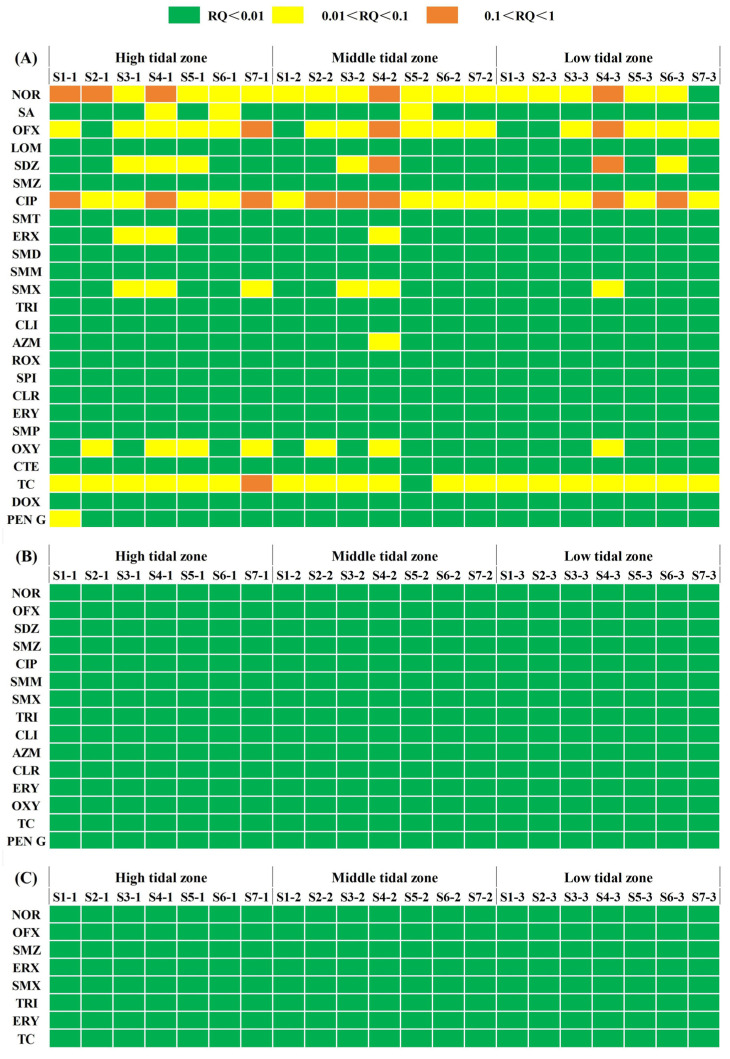
Risk quotients (RQs) of antibiotics in the sediment of Lianzhou Bay to algae (**A**), invertebrates (**B**), and fish (**C**).

The QUs and TCs showed the highest ecological risk, followed by SAs, while PCs and MLs showed the lowest ecological risk (Figure 3). Some studies have found that QUs and TCs in the sediments of Wangyang River [75], Laizhou Bay [76], and Beibu Gulf [77] have high ecological risks to algae. Furthermore, the QUs and TCs are easily accumulated by mangrove sediments due to their low solubility, bioavailability, and biodegradability [78,79]. In the study area, 11 kinds of antibiotics (NOR, OFX, SDZ, SMZ, CIP, ERX, SA, LOM, SMD, SMX, AZM, OXY, TC, and PEN G) with potential ecological risks to current algae were identified. The proportion of risk sites for CIP, TC, NOR, and OFX was 100%, 95%, 95%, and 81%, respectively, while the proportions of risk sites for other antibiotics were all less than 40% (Figure 3). This implied that CIP, TC, NOR, and OFX exhibited a higher risk to algae than the other kinds of antibiotics. The mangrove sediments could absorb large amounts of antibiotics due to their high organic carbon properties [13,80]. This result implied that the mangrove sediments with a high TOC concentration are more likely to accumulate antibiotics. CLI, ERY, SMM, and TRI had the lowest ecological risk among the 25 detected antibiotics. Although these antibiotics did not show high ecological risks, the degradation rates of these antibiotics were relatively low [81,82,83], resulting in their long-term existence in the mangrove environment. 

Additionally, the combined risk of multiple antibiotics can increase via synergistic effects, which need more attention [84]. The RQ_sum_-algae values were selected as the indicator of ecological risk in this study due to its greater sensitivity to antibiotics than fish and invertebrates. The RQ_sum_-algae varied from 0.09 to 0.98 (Figure S1), indicating that 95% of the sites were categorized as intermediate risk, with only site S5-2 being classified as low risk. These results imply that the ecological risk posed by antibiotics to the mangrove area is generally at a medium risk level. The mean value of RQ_sum_-algae in the high tidal zone was slightly higher than that in the middle tidal zone, and the mean value of RQ_sum_-algae in the low tidal zone was the lowest. The Spearman correlation analysis between logarithmic transformed RQ_sum_-algae and environmental factors showed that only pH was significantly negatively correlated with RQ_sum_-algae (*p* < 0.01), while the TOC, NO_3_−-N, NO_2_−-N, NH_4_^+^-N, and particle size were significantly positively correlated with RQ_sum_-algae (*p* < 0.01) (Table S4). The RQ_sum_-algae in the high tidal zone was slightly higher than that in the middle tidal zone, and the lowest in the low tidal zone. The high tidal zone impacted by land-based pollution exhibited the highest ecological risk, primarily due to significant antibiotic settlement. Previous studies have suggested that higher TOC and larger sediment particle size might contribute to the significant accumulation of antibiotics [33,34,39,46]. Therefore, the middle tidal zone characterized by higher levels of TOC and larger particle sizes showed ecological risk levels comparable to those in the high tidal zone. It is reported that the frequent tidal inundation in the low tidal zone could lead to a certain dilution effect on the concentration of some antibiotics in mangrove sediments [31], resulting in a low ecological risk of antibiotics in this zone. Notably, the higher the antibiotic risk level, the greater the probability of the generation and spread of drug-resistant bacteria and drug-resistant genetic elements, eventually leading to ARG pollution. However, the relatively stable ARGs could rapidly migrate and spread in the marine environment, posing a great potential threat to marine ecology and human health [9].

## 3. Materials and Methods

### 3.1. Sampling Sites and Sample Collection

**Figure 4:**
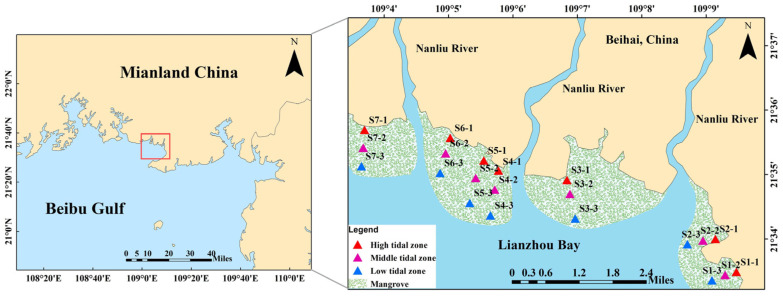
Location of sampling sites in the mangrove sediments of Lianzhou Bay, China.

The sediment samples were collected from 21 sites (3 parallel samples were collected from each site) in the high tidal zone, middle tidal zone, and low tidal zone in the mangrove area of Lianzhou Bay (108°58′00″–109°02′35″ E, 21°26′20″–21°37′00″ N), China, in April 2023 (Figure 4). The sites in the high tidal zone were all located near the effluent outlet of the aquaculture area (Figure S2). The surface sediment samples (depth < 5 cm) were obtained using a stainless-steel grab and stored in sterile polyethylene (PE) bags. These samples were immediately transported on ice via a portable refrigerator to the laboratory. Once back in the laboratory, the sediments were immediately freeze-dried by CHRIST Alpha 1-4 LSCbasic (Osterode, Germany), and then grounded and homogenized. Finally, the homogenized sediments were stored at −20 °C until analysis.

### 3.2. Materials and Solvents

A total of 39 antibiotics belonging to 5 classes, including 16 Sulfonamides (SAs), 11 Fluoroquinolones (QUs), 5 Tetracyclines (TCs), 6 Macrolides (MLs), and 1 Penicillin (PCs) were analyzed in this study. Trimethyl-^13^C_3_ caffeine was used as a surrogate standard to evaluate the antibiotic recoveries in sediments and simetone was used as an internal standard to calculate the possible interference of sediment matrix and instrumental analysis. All the antibiotic standards, Citrate buffer (pH 5), and EDTA buffer were purchased from Aladdin (Shanghai, China), and the trimethyl-^13^C_3_ caffeine and simetone standards were purchased from Dr. Ehrenstorfer GmbH (Oakville, ON, Canada) and Sigma-Aldrich (St. Louis, MO, USA), respectively. HPLC grade methanol and acetonitrile were obtained from Merck (Darmstadt, Germany). Formic acid was supplied by CNW (Dusseldorf, Germany). Disodium edetate dihydrate (Na_2_EDTA) was acquired from J&K^®^ (Beijing, China). Detailed information about the antibiotics, reagents, and solvents is provided in Text S1 and Table S5.

### 3.3. Analysis of the Physicochemical Parameters of the Sediments

The sediment was mixed with CO_2_-free deionized water at a volume ratio of 1:2.5, and the pH of the sediment was measured using a pH meter (Mettler-Toledo, Greifensee, Switzerland) [85]. The TOC in the freeze-dried sediment was analyzed using a TOC analyzer (Vario TOC, Elementar, Langenselbold, Germany) [86]. The particle size of the sediment was measured using a Horiba LA-300 particle sizer (Horiba Group, Edison, NJ, USA). Exchangeable ammonium (NH_4_^+^-N), nitrate (NO_3_^−^-N) and nitrite (NO_2_^−^-N) were extracted from the fresh sediments using 2 M of KCl and quantified spectrophotometrically on a continuous flow analyzer (SAN Plus, Skalar Analytical B.V., Breda, The Netherlands) with detection limits of 0.5 M for NH_4_^+^-N and 0.1 M for NO_3_^−^-N and NO_2_^−^-N [87]. Detailed data about the physicochemical properties of mangrove sediments are shown in Table S6.

### 3.4. Quantitative Analysis of Antibiotics in Sediments

Various physicochemical properties of the wide family of antibiotics, together with matrix interference, make their reliable analysis in complex environmental samples (e.g., sediment and water) very challenging [88,89]. The preparation of sediment samples was conducted as per the methodology outlined in a previous study. Briefly, 2 g of freeze-dried and homogenized sediment samples was weighted into a PTEE tube (50 mL), and 50 ng of ^13^C_3_-caffeine and 0.3 g NaF were spiked as a surrogate and ion exchanger, respectively. Ultrasound-assisted extraction using 30 mL extraction solution, including 15 mL of methanol, 10 mL of citrate buffer (pH 5), and 5 mL of 0.1 M EDTA buffer, was then conducted for 20 min, after which the extract was centrifuged for 10 min at 5000 rpm and the upper supernant was transferred into a clean glass jar. The same extraction process was repeated twice, and all the obtained supernants were combined and diluted to 500 mL with ultrapure water. The diluted extract was acidified to pH 5.0 by adding drops of 6 mol/L HCl, and then 0.8 g of Na_2_EDTA was added as the chelating agent, after which the adjusted extract was loaded onto pre-conditioned (5 mL methanol) and pre-equilibrated (5 mL ultrapure water) Oasis HLB cartridges (6 mL, 500 mg; Waters), with a flow rate of 5–10 mL/min. Following that, 5 mL methanol and 5 mL dichloromethane were used to elute the antibiotics in the cartridge, and then the eluent was condensed to near dryness under a gentle nitrogen flow at room temperature. Finally, the concentrated eluate was diluted to an equal volume of 1.0 mL with methanol and 20 μL of a simetone (100 μg/L) was added as internal standard.

The target antibiotics were analyzed by the Dionex series high-performance liquid chromatograph system coupled to an AB Science triple quadrupole mass spectrometer (Palo Alto, CA, USA), equipped with an electrospray ionization (ESI) source in a multiple reaction monitoring (MRM) mode. The selected antibiotics were separated using an Agilent (Santa Clara, CA, USA) ZORBAX Eclipse Plus C18 (150 mm × 2.1 mm, 3.5 μm) column, and the column temperature was at 40 °C. The 0.2% formic acid in ultrapure water (eluent A) and acetonitrile (eluent B) were used as the mobile phase. The elution program was as follows: 1% B (0–5 min), 10% B (5–25 min), 50% B (25–26 min), and 1% B (26–30 min), and the flow rate was at 0.3 mL/min. All the targets were analyzed in the ESI+ mode, the ion source temperature was 550 °C, the ion spray voltage was 500 V, the curtain gas was 35 kPa, and the ion source gas was 60 kPa.

### 3.5. Quality Assurance and Quality Control (QA/QC)

The experimental procedures were subjected to strict QA/QC procedures. All the glass containers were rinsed three times with ultrapure water and methanol, respectively, and then baked at 450 °C for 4 h in a muffle furnace. The concentrations of the antibiotic were quantified using the internal standard method. Linearity was evaluated using an 8-point calibration curve (1 ppb, 2 ppb, 5 ppb, 10 ppb, 20 ppb, 50 ppb, 100 ppb, and 200 ppb) for each compound (R^2^ > 0.99). The limit of detection (LOD) (0.02–1.09 ng/g for sediment samples) and limit of quantitation (LOQ) (0.04–1.75 ng/g for sediment samples) were determined as the minimum detectable amount of an analyte with signal-to-noise (S/N) ratios of 3 and 10, respectively. Procedural blanks and standard solutions were included in each batch of 5 samples to evaluate the possible interference or background contamination. The concentrations of all compounds in the procedural blank were below the detection limit. The spiked recoveries of the target antibiotics in sediments ranged from 55 to 94% with relative standard deviations (RSD) lower than 20%. The recovery of ^13^C_3_-caffeine surrogate in sediment samples was 74–93%. 

### 3.6. Ecological Risk Assessment

The potential ecological risks associated with target antibiotics were evaluated by risk quotients (RQ_s_), using Equations (1)–(6) [1,90,91,92,93]. The risk levels were divided into four categories, i.e., insignificant risk (RQ < 0.01), low risk (0.01 ≤ RQ < 0.1), medium risk (0.1 ≤ RQ < 1), and high risk (RQ > 1) [94].

PNEC_water_ = EC_50_(LC_50_)/AF (1)

PNEC_Sediment_ = PNEC_water_ × *K*_d_ (2)

*K*_oc_ = *K*_d_ × 100/*f_oc_* (3)

log*K*_oc_ = 0.529log*K*_ow_ + 1.082 (4)

RQ_i_ = MEC_sediment_/PNEC_Sediment_ (5)

RQ_sum_ = ƩRQ_i_ (6)

where PNEC_water_ denotes the predicted no-effect concentration (PNEC means a concentration that does not normally produce adverse effects) of antibiotics in water, μg/L, EC_50_ denotes the half-maximal effective mass concentration, and LC_50_ denotes the half-lethal mass concentration, mg/L. Episodes of toxicity within one day were considered acute and those greater than one day were considered chronic. AF denotes the assessment factor and takes a value of 1000 when using acute toxicity data and 100 when using chronic toxicity data [95]. PNEC_Sediment_ denotes the predicted non-effect content of antibiotics in soil, mg/kg. *K*_oc_ means the organic carbon partition coefficient of the antibiotic, and *K*_d_ is their sediment-water distribution coefficients. The *f_oc_* (%) is the TOC concentration in the sediment; *K*_oc_ can be calculated using an octanol-water partition coefficient (log*K*_ow_). MEC_sediment_ means the measured antibiotic concentrations in sediment, μg/g. The total RQ_sum_ in a sediment sample was caculated by summing the RQ of each individual antibiotic together. EC_50_ or LC_50_ were obtained from previous literature or the ECOTOX Drugbank Database (https://go.drugbank.com/, accessed on 1 December 2023) (Table S7). SD, MBX, and PFLX were not found EC_50_ or LC_50_ data.

### 3.7. Statistical Analysis

The averages and standard deviations were computed using Excel 2016 (Microsoft, Redmond, WA, USA), and the data were visualized using Origin 2018 (OriginLab, Northampton, MA, USA). The relationship between the spatial distribution of antibiotics and various environmental factors was assessed through a multivariate analysis, including a detrended correspondence analysis (DCA) and a redundancy analysis (RDA), using R 4.2.2 (R Development Core Team, Vienna, Austria) [96]. The length of the first ordination gradient was determined using DCA, and if the calculated value was less than 3, RDA was selected for further analysis of the dataset. Additionally, the sources of the antibiotics in the sediment samples were identified through a varimax-rotated component matrix analysis using SPSS 26 (IBM Analytics, Armonk, NY, USA) [97].

## 4. Conclusions

In this study, the occurrence, distribution, potential source, and risk assessment of 39 antibiotics in mangrove sediments from Lianzhou Bay, China, were investigated. The concentrations of these five classes of antibiotics were as follows: SAs > TCs > QUs > PCs > MLs. The spatial distribution of the antibiotic concentrations varied as high tidal zone > middle tidal zone > low tidal zone. Additionally, the spatial distribution of antibiotics in mangrove sediments was significantly correlated (*p* < 0.05) with TOC, pH, NO_3_^−^-N, and NO_2_^−^-N. The source analysis indicated that untreated sewage from aquaculture activities constituted the primary source of antibiotics in the local mangrove environment. The risk assessment indicated that low to medium risks posed by an individual antibiotic were only found for algae, among which CIP, TC, NOR, and OFX could pose relatively higher risks to algae. Based on the RQ_sum_-algae results, 95% of the sites were classified as at the intermediate risk level. The overall ecological risk level of antibiotics in the middle tidal zone was slightly lower than that in the high tidal zone, and the lowest in the low tidal zone. Effective regulation of discharges, particularly from aquaculture, is pivotal for managing antibiotic risks in Lianzhou Bay.

## Figures and Tables

**Table 1:** Physicochemical properties of mangrove sediments in Lianzhou Bay.

	High Tidal Zone	Middle Tidal Zone	Low Tidal Zone
pH	6.85 ^a^ ± 0.16	7.14 ^b^ ± 0.15	7.15 ^b^ ± 0.22
NO_3_−-N(μmol/L)	1.64 ^a^ ± 0.54	1.31 ^ab^ ± 0.39	1.09 ^b^ ± 0.19
NO_2_−-N (μmol/L)	2.0 × 10^−2^ ± 0.6 × 10^−3^	2.2 × 10^−2^ ± 9.0 × 10^−3^	1.9 × 10^−2^ ± 0.6 × 10^−3^
NH_4_^+^-N (μmol/L)	0.39 ± 0.14	0.50 ± 0.48	0.43 ± 0.34
TOC (%)	1.74 ± 0.57	1.68 ± 1.15	1.11 ± 0.58
Particle size (μm)	10.59 ± 5.75	25.52 ± 43.51	17.28 ± 13.51

Note: Values with different letters (a and b) indicate a significant difference at *p* < 0.05 among the tidal zones, and values with the same letter indicate that the difference was not significant at *p* > 0.05.

**Table 2:** Varimax-rotated component matrix following PCA of all sediment samples.

Antibiotics	Rotated Component Number ^a^				
	1	2	3	4	5
NOR	−0.321	−0.066	−0.819	−0.010	0.183
CIP	**0.778**	0.177	−0.190	−0.226	0.083
LOM	**0.812**	−0.112	0.287	−0.23	0.286
ERX	−0.304	0.118	0.167	**0.804**	−0.222
OFX	**0.668**	0.458	0.330	0.107	−0.171
AZM	0.003	**0.556**	0.018	**0.646**	−0.366
ROX	**0.689**	0.093	0.377	0.268	0.190
CLI	−0.58	−0.404	−0.142	−0.302	0.162
ERY	0.288	**0.731**	−0.023	0.401	0.111
SA	0.145	**0.817**	0.090	−0.092	0.425
SPD	**0.754**	0.307	**0.528**	0.061	0.102
SDZ	0.249	0.389	**0.511**	**0.676**	−0.038
SMX	**0.585**	−0.454	**0.530**	0.104	−0.053
SMZ	−0.045	**0.872**	0.230	0.171	−0.046
SMT	−0.259	−0.291	−0.783	−0.369	0.151
SMD	0.022	−0.048	0.161	**0.914**	0.170
SMM	0.500	**0.574**	0.392	−0.388	0.131
OXY	**0.603**	0.197	0.253	−0.007	0.250
CTE	**0.648**	0.073	0.305	−0.008	**0.560**
TC	**0.715**	−0.411	−0.052	−0.095	−0.351
DOX	0.123	0.138	−0.254	−0.072	**0.839**
PEN G	−0.041	−0.103	−0.884	−0.206	−0.067
Percentage variance explained (%)	24.663	17.391	17.127	14.501	8.555

^a^ Absolute value > 0.5 are highlighted.

## Data Availability

The data presented in this study are available on request from the corresponding author.

## Supplementary Materials

The following supporting information can be downloaded at: https://www.mdpi.com/article/10.3390/antibiotics13090820/s1. Refs. [98,99,100,101,102,103,104,105,106,107,108,109,110,111,112,113,114,115,116,117,118,119,120,121,122,123,124] are cited in Supplementary Materials. Text S1: Materials and solvents; Table S1: The antibiotic concentrations and detection frequencies in sediments (ng/g dw); Table S2: Pearson analysis of antibiotics (Detection frequency >10 %); Table S3: Estimated RQsum values of algae, invertebrate, and fish in mangrove sediments of Lianzhou Bay; Table S4: The correlation between antibiotics, RQsum, and environmental factors in mangrove sediments; Table S5: Basic information on tested antibiotics; Table S6: Physicochemical properties of mangrove sediments in Lianzhou Bay; Table S7: The parameters of detected antibiotics for ecological risk assessment; Figure S1: Estimated RQsum-algae values of target antibiotics in mangrove sediments of Lianzhou Bay; Figure S2: Location of sampling sites and the aquaculture area in the mangrove sediments of Lianzhou Bay, China.
