# Supplementary material for: Occurrence, Source Apportionment, and Risk Assessment of Antibiotics in Mangrove Sediments from the Lianzhou Bay, China
Source: Antibiotics (Basel). 2024 Aug 28;13(9):820. doi: 10.3390/antibiotics13090820 (PMC11429403; doi:10.3390/antibiotics13090820)
Supplement: Supplementary file 1 [file antibiotics-13-00820-s001.zip › Supplementary materials.docx]

**Occurrence,** **source apportionment, and risk assessment of antibiotics** **in mangrove sediments from the Lianzhou Bay****, China**

Pengfei Sun^a,b^, Yongyu Tan^a,b^, Zuhao Zhu^a,b^, Tinglong Yang^a,b^, Li Zhang^a,b,^*

^a^ Guangxi Beibu Gulf Key Laboratory of Marine Resources, Environment and Sustainable Development, Fourth Institute of Oceanography, Ministry of Natural Resources, Beihai 536000, China

^b^ Key Laboratory of Tropical Marine Ecosystem and Bioresource, Fourth Institute of Oceanography, Ministry of Natural Resources, Beihai 536000, China

***Correspondence**: zhangli@4io.org.cn

Number of pages: 15

Number of texts: 1

Number of tables: 7

Number of figures: 2

**Text S1** Materials and solvents

Norfloxacin (NOR, ≥ 98%), Ciprofloxacin (CIP, ≥ 98%), Enrofloxacin (ERX, ≥ 98%), Enoxacin (ENX, ≥ 98%), Marbofloxacin (MBX, ≥ 98%), Ofloxacin (OFX, ≥ 98%), Sulfacetamide (SA, ≥ 98%), Sulfapyridine (SPD, ≥ 98%), Sulfadiazine (SDZ, ≥ 98%), Sulfamethoxazole (SMX, ≥ 98%), Sulfamerazine (SMZ, ≥ 98%), Sulfamethizole (SMT, ≥ 98%), Sulfamethazine (SM2, ≥ 98%), Sulfamonomethoxine (SMM, ≥ 98%), Sulfadoxine (SD, ≥ 98%), Sulfamethoxypyrimidine (ST, ≥ 98%), Trimethoprim (TRI, ≥ 98%), Sulfaisoxazole (SOX, ≥ 98%), and Sulfamethoxypy ridazine (SMP, ≥ 98%) were purchased from Supelco (PA, USA). Clarithromycin (CLR, ≥ 98%), and Lincomycin (LIN, ≥ 96%) were purchased from Sigma–Aldrich (St. Louis, MO, USA).Lomefloxacin (LOM, ≥ 98%), Fleroxacin (MBX, ≥ 98%), Pefloxacin (PFLX, ≥ 98%), Azithromycin (AZM, ≥ 98%), Roxithromycin (AZM, ≥ 98%), Spiramycin (SPI, ≥ 98%), Clindamycin (CLI, ≥ 99%), Tylosin (TLS, ≥ 98%), Erythromycin (ERY, ≥ 98%), Sulfabenzamide (SBD, ≥ 99%), Sulfamethoxine (SMD, ≥ 98%), Sulfadimethoxine (SDM, ≥ 98%), Oxytetracycline (OXY, ≥ 98%), Chlortetracyelin (CTE, ≥ 98%), Tetracycline (TC, ≥ 98%), Metacycline (MET, ≥ 99%), Doxycycline (DOX, ≥ 99%), and Penicillin G (PEN G, ≥ 98%) were purchased from Aladdin (Shanghai, China).

Ultra-pure water was prepared with a Milli-Q water purification system (Millipore, Bedford, Massachusetts, USA).

.

**Table S1** The antibiotic concentrations and detection frequencies in sediments (ng/g dw).

| **Compounds** | **Antibiotic concentrations (ng/g dw)** | | | **Detection frequencies** |
| --- | --- | --- | --- | --- |
|  | **Max** | **Min** | **Mean** (±SD) |  |
| NOR | 18.73 | 3.01 | 9.95 ± 4.53 | 100% |
| CIP | 18.45 | 1.88 | 10.35 ± 3.94 | 100% |
| LOM | 5.08 | ND | 0.80 ± 1.40 | 52.38% |
| ERX | 8.41 | ND | 3.80 ± 2.88 | 95.24% |
| MBX | 0.97 | ND | 0.10 ± 0.26 | 0.00% |
| PFLX | 0.20 | ND | 0.01 ± 0.04 | 0.00% |
| ENX | ND | ND | ND | 0.00% |
| FLX | ND | ND | ND | 0.00% |
| OFX | 13.64 | 0.38 | 5.68 ± 4.01 | 100% |
| AZM | 11.97 | ND | 3.84 ± 3.66 | 95.24% |
| ROX | 5.09 | ND | 1.77 ± 1.55 | 90.48% |
| QUs | 51.10 | 18.53 | 36.18 ± 3.89 | 100% |
| SPI | 0.43 | ND | 0.03 ± 0.1 | 9.52% |
| CLI | 1.23 | ND | 0.20 ± 0.32 | 38.10% |
| CLR | 0.36 | ND | 0.08 ± 0.23 | 14.29% |
| LIN | ND | ND | ND | 0.00% |
| TLS | ND | ND | ND | 0.00% |
| ERY | 2.53 | ND | 1.01 ± 0.77 | 95.24% |
| MLs | 2.89 | 0.58 | 1.31 ± 0.39 | 100% |
| SA | 8.14 | ND | 1.63 ± 1.95 | 80.95% |
| SPD | 32.56 | 1.73 | 15.96 ± 8.52 | 100% |
| SDZ | 19.89 | 0.17 | 11.17 ± 6.07 | 100% |
| SMX | 21.93 | 1.48 | 9.23 ± 5.15 | 100% |
| SMZ | 24.22 | 3.45 | 11.59 ± 5.82 | 100% |
| SMT | 1.75 | ND | 0.28 ± 0.50 | 33.33% |
| SBD | ND | ND | ND | 0.00% |
| SM2 | ND | ND | ND | 0.00% |
| SMD | 39.03 | 5.71 | 15.10 ± 7.56 | 100% |
| SMM | 2.35 | ND | 0.61 ± 0.75 | 57.14% |
| SDM | ND | ND | ND | 0.00% |
| SD | 0.19 | ND | 0.04 ± 0.70 | 0.00% |
| ST | ND | ND | ND | 0.00% |
| TRI | 1.05 | 0 | 0.08 ± 0.26 | 9.52% |
| SOX | ND | ND | ND | 0.00% |
| SMP | ND | ND | ND | 0.00% |
| SAs | 100.07 | 15.25 | 65.65 ± 6.11 | 100% |
| OXY | 12.33 | 0.70 | 6.45 ± 3.19 | 100% |
| CTE | 28.74 | 4.26 | 14.83 ± 6.56 | 100% |
| TC | 13.93 | 1.5 | 6.01 ± 2.94 | 100% |
| MET | ND | ND | ND | 0.00% |
| DOX | 15.41 | 6.93 | 11.45 ± 2.58 | 100% |
| TCs | 65.33 | 20.74 | 38.75 ± 5.67 | 100% |
| PEN G | 7.40 | ND | 0.84 ± 1.67 | 42.86% |
| PCs | 7.40 | ND | 0.84 ± 1.67 | 42.86% |
| ∑Antibiotics | 202.24 | 65.57 | 142.73 ± 36.76 | 50.06% |

**Note: ND means not detected.**

**Table S2** Pearson analysis of antibiotics (Detection frequency >10 %)

|  | NOR | CIP | LOM | ERX | OFX | AZM | ROX | CLI | ERY | SA | SPD | SDZ | SMX | SMZ | SMT | SMD | SMM | OXY | CTE | TC | DOX | PEN G |
| --- | --- | --- | --- | --- | --- | --- | --- | --- | --- | --- | --- | --- | --- | --- | --- | --- | --- | --- | --- | --- | --- | --- |
| NOR | 1 |  |  |  |  |  |  |  |  |  |  |  |  |  |  |  |  |  |  |  |  |  |
| CIP | -0.167 | 1 |  |  |  |  |  |  |  |  |  |  |  |  |  |  |  |  |  |  |  |  |
| LOM | -0.405 | 0.582** | 1 |  |  |  |  |  |  |  |  |  |  |  |  |  |  |  |  |  |  |  |
| ERX | -0.146 | -0.394 | -0.458* | 1 |  |  |  |  |  |  |  |  |  |  |  |  |  |  |  |  |  |  |
| OFX | -0.470* | 0.565** | 0.542* | -0.03 | 1 |  |  |  |  |  |  |  |  |  |  |  |  |  |  |  |  |  |
| AZM | -0.057 | -0.084 | -0.262 | 0.644** | 0.469* | 1 |  |  |  |  |  |  |  |  |  |  |  |  |  |  |  |  |
| ROX | -0.525* | 0.423 | 0.600** | 0.125 | 0.496* | 0.061 | 1 |  |  |  |  |  |  |  |  |  |  |  |  |  |  |  |
| CLI | 0.419 | -0.26 | -0.325 | -0.043 | -0.587** | -0.435* | -0.484* | 1 |  |  |  |  |  |  |  |  |  |  |  |  |  |  |
| ERY | -0.074 | 0.237 | 0.072 | 0.332 | 0.461* | 0.536* | 0.568** | -0.494* | 1 |  |  |  |  |  |  |  |  |  |  |  |  |  |
| SA | -0.147 | 0.288 | 0.25 | -0.05 | 0.405 | 0.222 | 0.269 | -0.343 | 0.615** | 1 |  |  |  |  |  |  |  |  |  |  |  |  |
| SPD | -0.661** | 0.564** | 0.742** | -0.032 | 0.838** | 0.216 | 0.770** | -0.551** | 0.442* | 0.442* | 1 |  |  |  |  |  |  |  |  |  |  |  |
| SDZ | -0.543* | 0.046 | 0.112 | 0.621** | 0.592** | 0.685** | 0.532* | -0.564** | 0.571** | 0.318 | 0.639** | 1 |  |  |  |  |  |  |  |  |  |  |
| SMX | -0.616** | 0.349 | 0.630** | 0.021 | 0.419 | -0.144 | 0.556** | -0.103 | -0.117 | -0.301 | 0.630** | 0.355 | 1 |  |  |  |  |  |  |  |  |  |
| SMZ | -0.279 | 0.069 | -0.107 | 0.293 | 0.509* | 0.596** | 0.079 | -0.398 | 0.674** | 0.656** | 0.365 | 0.571** | -0.2 | 1 |  |  |  |  |  |  |  |  |
| SMT | 0.711** | 0.092 | -0.31 | -0.34 | -0.652** | -0.491* | -0.534* | 0.599** | -0.392 | -0.237 | -0.685** | -0.805** | -0.417 | -0.488* | 1 |  |  |  |  |  |  |  |
| SMD | -0.179 | -0.167 | -0.099 | 0.659** | 0.157 | 0.478* | 0.291 | -0.331 | 0.278 | -0.003 | 0.137 | 0.669** | 0.167 | 0.161 | -0.427 | 1 |  |  |  |  |  |  |
| SMM | -0.518* | 0.504* | 0.521* | -0.294 | 0.600** | 0 | 0.534* | -0.445* | 0.417 | 0.670** | 0.769** | 0.301 | 0.188 | 0.453* | -0.4 | -0.296 | 1 |  |  |  |  |  |
| OXY | -0.286 | 0.419 | 0.611** | -0.229 | .434* | 0.076 | 0.699** | -0.389 | 0.435* | 0.3 | 0.660** | 0.331 | 0.336 | 0.055 | -0.393 | 0.008 | 0.544* | 1 |  |  |  |  |
| CTE | -0.348 | 0.457* | 0.738** | -0.269 | .475* | -0.162 | 0.603** | -0.439* | 0.248 | 0.419 | 0.723** | 0.32 | 0.460* | 0.098 | -0.346 | 0.161 | 0.603** | 0.487* | 1 |  |  |  |
| TC | -0.211 | 0.35 | 0.585** | -0.3 | 0.315 | -0.175 | 0.27 | -0.402 | -0.201 | -0.341 | 0.322 | -0.075 | 0.519* | -0.346 | -0.107 | -0.115 | 0.074 | 0.102 | 0.28 | 1 |  |  |
| DOX | 0.307 | 0.246 | 0.292 | -0.36 | -0.014 | -0.223 | -0.01 | 0.012 | 0.1 | 0.442* | 0.096 | -0.104 | -0.154 | 0.104 | 0.241 | 0.04 | 0.13 | 0.117 | 0.481* | -0.142 | 1 |  |
| PEN G | 0.633** | 0.159 | -0.27 | -0.206 | -.458* | -0.227 | -0.388 | 0.276 | -0.151 | -0.145 | -0.520* | -0.611** | -0.406 | -0.314 | 0.856** | -0.338 | -0.286 | -0.295 | -0.327 | 0.064 | 0.118 | 1 |

Note: * * indicates a very significant correlation (*p* <0.01); * indicates a significant correlation (*p* < 0.05).

**Table S3** Estimated RQ_sum_ values of algae, invertebrate, and fish in mangrove sediments of Lianzhou Bay

| **Site** | **RQ_sum_-****algae** | **RQ_sum_-invertebrate** | **RQ_sum_-fish** |
| --- | --- | --- | --- |
| S1-1 | 4.65E-01 | 3.45E-03 | 6.82E-05 |
| S2-1 | 3.04E-01 | 3.81E-03 | 4.42E-05 |
| S3-1 | 2.60E-01 | 3.37E-03 | 9.55E-05 |
| S4-1 | 5.37E-01 | 3.85E-03 | 1.06E-04 |
| S5-1 | 2.57E-01 | 1.47E-03 | 3.50E-05 |
| S6-1 | 2.42E-01 | 9.65E-04 | 3.66E-05 |
| S7-1 | 6.28E-01 | 5.15E-03 | 1.29E-04 |
| S1-2 | 1.25E-01 | 1.04E-03 | 1.99E-05 |
| S2-2 | 2.60E-01 | 2.13E-03 | 4.27E-05 |
| S3-2 | 3.20E-01 | 3.54E-03 | 7.87E-05 |
| S4-2 | 9.81E-01 | 5.93E-03 | 1.55E-04 |
| S5-2 | 9.51E-02 | 3.86E-03 | 1.13E-04 |
| S6-2 | 1.87E-01 | 7.51E-04 | 2.79E-05 |
| S7-2 | 1.69E-01 | 1.59E-03 | 4.08E-05 |
| S1-3 | 1.46E-01 | 2.09E-03 | 3.81E-05 |
| S2-3 | 1.46E-01 | 1.71E-03 | 2.74E-05 |
| S3-3 | 1.77E-01 | 1.67E-03 | 4.56E-05 |
| S4-3 | 4.90E-01 | 3.01E-03 | 9.37E-05 |
| S5-3 | 1.39E-01 | 8.18E-04 | 3.22E-05 |
| S6-3 | 2.71E-01 | 1.57E-03 | 5.15E-05 |
| S7-3 | 1.43E-01 | 1.29E-03 | 3.01E-05 |

**Table S4** The correlation between antibiotics, RQ_sum_, and environmental factors in mangrove sediments.

|  | pH | NO_3_^－^-N | NO_2_^－^-N | NH_4_^+^-N | TOC | particle size |
| --- | --- | --- | --- | --- | --- | --- |
| QUs | -0.416 | 0.361 | -0.138 | **0.457*** | 0.299 | 0.319 |
| MLs | -0.274 | 0.234 | -0.075 | 0.379 | 0.181 | 0.029 |
| SAs | **-0.600**** | 0.266 | -0.351 | 0.212 | 0.221 | 0.109 |
| TCs | -0.343 | 0.126 | -0.392 | 0.013 | -0.143 | 0.043 |
| PCs | 0.326 | -0.013 | 0.200 | 0.047 | -0.255 | -0.107 |
| ∑Antibiotics | -0.544* | 0.270 | -0.388 | 0.243 | 0.196 | 0.133 |
| RQ_sum_-algae | **-0.644**** | **0.575**** | **0.475*** | **0.716**** | **0.691**** | 0.301 |
| RQ_sum_-invertebrate | -0.292 | **0.590**** | **0.473*** | 0.327 | **0.875**** | 0.376 |
| RQ_sum_-fish | **-0.577**** | **0.573**** | 0.286 | 0.316 | **0.891**** | 0.292 |

** At the *p*＜0.01 level (two-tailed), the correlation is significant.

* At the *p*＜0.05 level (two-tailed), the correlation was significant.

**Table S5** Basic information on tested antibiotics

| Species | Name | Abbreviation | Molecular formula | CAS number | Molecular Weight(g/mol) |
| --- | --- | --- | --- | --- | --- |
| Fluoroquinolones  (QUs) | Norfloxacin | NOR | C_16_H_18_FN_3_O_3_ | 70458-96-7 | 319.33 |
|  | Ciprofloxacin | CIP | C_17_H_18_FN_3_O_3_ | 85721-33-1 | 331.34 |
|  | Lomefloxacin | LOM | C_17_H_19_F_2_N_3_O_3_·HCl | 98079-52-8 | 387.81 |
|  | Enrofloxacin | ERX | C_19_H_22_N_3_O_3_F | 93106-60-6 | 359.39 |
|  | Enoxacin | ENX | C_15_H_17_FN_4_O_3_ | 74011-58-8 | 347.34 |
|  | Marbofloxacin | MBX | C_17_H_19_N_4_O_4_F | 115550-35-1 | 362.36 |
|  | Fleroxacin | FLX | C_17_H_18_F_3_N_3_O_3_ | 799660-72-3 | 369.34 |
|  | Ofloxacin | OFX | C_18_H_20_FN_3_O_4_ | 82419-36-1 | 361.37 |
|  | Pefloxacin | PFLX | C_17_H_20_N_3_O_3_F·CH_4_O_3_S | 149676-40-4 | 465.49 |
|  | Azithromycin | AZM | C_38_H_72_N_2_O_12_ | 83905-01-5 | 748.98 |
|  | Roxithromycin | ROX | C_41_H_76_N_2_O_15_ | 80214-83-1 | 837.05 |
| Macrolides  (MLs) | Spiramycin | SPI | C_43_H_74_N_2_O_14_ | 8025-81-8 | 843.06 |
|  | Clindamycin | CLI | C_18_H_34_C_12_N_2_O_5_S | 58207-19-5 | 479.46 |
|  | Clarithromycin | CLR | C_38_H_69_NO_13_ | 81103-11-9 | 747.95 |
|  | Lincomycin | LIN | C_18_H_34_N_2_O_6_S·HCL | 859-18-7 | 443.00 |
|  | Tylosin | TLS | 2(C_46_H_77_NO_17_)·C_4_H_6_O_6_ | 74610-55-2 | 1982.31 |
|  | Erythromycin | ERY | C_37_H_67_NO_13_ | 114-07-8 | 733.93 |
| Sulfonamides  (SAs) | Sulfacetamide | SA | C_8_H_10_N_2_SO_3_ | 144-80-9 | 214.24 |
|  | Sulfapyridine | SPD | C_11_H_11_N_3_О_2_S | 144-83-2 | 249.29 |
|  | Sulfadiazine | SDZ | C_10_H_10_N_4_O_2_S | 68-35-9 | 250.28 |
|  | Sulfamethoxazole | SMX | C_10_H_11_N_3_O_3_S | 723-46-6 | 253.28 |
|  | Sulfamerazine | SMZ | C_11_H_12_N_4_O_2_S | 127-79-7 | 264.30 |
|  | Sulfamethizole | SMT | C_9_H_10_N_4_O_2_S_2_ | 144-82-1 | 270.33 |
|  | Sulfabenzamide | SBD | C_13_H_12_N_2_O_3_S | 127-71-9 | 276.31 |
|  | Sulfamethazine | SM2 | C_12_H_14_N_4_O_2_S | 57-68-1 | 278.33 |
|  | Sulfamethoxine | SMD | C_11_H_12_N_4_O_3_S | 651-06-9 | 280.30 |
|  | Sulfamonomethoxine | SMM | C_11_H_12_N_4_O_3_S | 1220-83-3 | 280.30 |
|  | Sulfadoxine | SD | C_12_H_14_N_4_O_4_S | 2447-57-6 | 310.33 |
|  | Sulfadimethoxine | SDM | C_12_H_13_N_4_NaO_4_S | 1037-50-9 | 332.31 |
|  | Sulfamethoxypyrimidine | ST | C_9_H_9_N_3_O_2_S_2_ | 72-14-0 | 255.32 |
|  | Trimethoprim | TRI | C_14_H_18_N_4_O_3_ | 738-70-5 | 290.32 |
|  | Sulfaisoxazole | SOX | C_11_H_13_N_3_O_3_S | 127-69-5 | 267.30 |
|  | Sulfamethoxypy ridazine | SMP | C_11_H_12_N_4_O_3_S | 80-35-3 | 280.30 |
| Tetracyclines  (TCs) | Oxytetracycline | OXY | C_22_H_24_N_2_O_9_·HCl | 2058-46-0 | 496.89 |
|  | Chlortetracyelin | CTE | C_22_H_23_ClN_2_O_8_·HCl | 64-72-2 | 515.34 |
|  | Tetracycline | TC | C_22_H_24_N_2_O_8_·HCl | 64-75-5 | 480.9 |
|  | Metacycline | MET | C_22_H_22_N_2_O_8_·HCl | 3963-95-9 | 478.88 |
|  | Doxycycline | DOX | C_22_H_24_N_2_O_8_·HCl | 24390-14-5 | 512.94 |
| Penicillins  (PCs) | Penicillin G | PEN G | C_16_H_18_N_2_O_4_S | 61-33-6 | 334.39 |

**Table** **S6** Physicochemical properties of mangrove sediments in Lianzhou Bay

| Sample | pH | NO_3_^－^-N(μmol/L) | NO_2_^－^-N (μmol/L) | NH_4_^＋^-N (μmol/L) | TOC (%) | Particle size (μm) |
| --- | --- | --- | --- | --- | --- | --- |
| S1-1 | 6.90 | 1.51 | 2.28×10^-2^ | 0.52 | 1.69 | 7.79 |
| S2-1 | 7.03 | 1.38 | 2.80×10^-2^ | 0.43 | 1.68 | 9.48 |
| S3-1 | 6.59 | 1.53 | 2.03×10^-2^ | 0.14 | 1.78 | 7.77 |
| S4-1 | 6.78 | 2.84 | 2.11×10^-2^ | 0.46 | 2.87 | 10.12 |
| S5-1 | 6.98 | 1.56 | 1.94×10^-2^ | 0.54 | 1.33 | 8.53 |
| S6-1 | 6.69 | 1.29 | 1.32×10^-2^ | 0.28 | 1.05 | 7.02 |
| S7-1 | 6.96 | 1.36 | 1.16×10^-2^ | 0.35 | 1.76 | 23.40 |
| High tidal zone | 6.85 ± 0.16 | 1.64^a^ ± 0.54 | 2.0×10^-2^ ±0.6×10^-3^ | 0.39 ± 0.14 | 1.74 ± 0.57 | 10.59 ± 5.75 |
| S1-2 | 7.29 | 1.11 | 1.86×10^-2^ | 0.27 | 0.59 | 8.96 |
| S2-2 | 7.08 | 1.16 | 2.49×10^-2^ | 0.37 | 1.63 | 8.58 |
| S3-2 | 7.26 | 1.13 | 2.22×10^-2^ | 0.42 | 1.88 | 7.50 |
| S4-2 | 6.88 | 2.15 | 4.08×10^-2^ | 1.57 | 3.59 | 124.13 |
| S5-2 | 7.17 | 1.32 | 1.82×10^-2^ | 0.26 | 2.71 | 10.84 |
| S6-2 | 7.29 | 1.02 | 1.92×10^-2^ | 0.32 | 0.70 | 11.15 |
| S7-2 | 7.07 | 1.28 | 1.25×10^-2^ | 0.31 | 0.68 | 7.50 |
| Middle tidal zone | 7.14^b^ ± 0.15 | 1.31^ab^ ± 0.39 | 2.2×10^-2^ ± 9.0×10^-3^ | 0.50 ± 0.48 | 1.68 ± 1.15 | 25.52 ± 43.51 |
| S1-3 | 7.33 | 1.09 | 2.03×10^-2^ | 0.28 | 1.06 | 10.71 |
| S2-3 | 7.47 | 1.16 | 1.91×10^-2^ | 0.29 | 0.94 | 19.42 |
| S3-3 | 7.13 | 1.11 | 1.58×10^-2^ | 0.22 | 1.13 | 7.83 |
| S4-3 | 6.86 | 1.36 | 3.03×10^-2^ | 1.19 | 2.35 | 23.40 |
| S5-3 | 6.97 | 1.22 | 1.39×10^-2^ | 0.36 | 0.62 | 7.31 |
| S6-3 | 7.02 | 0.85 | 1.47×10^-2^ | 0.42 | 1.00 | 44.37 |
| S7-3 | 7.27 | 0.86 | 1.59×10^-2^ | 0.25 | 0.65 | 7.92 |
| Low tidal zone | 7.15^b^ ± 0.22 | 1.09^b^ ± 0.19 | 1.9×10^-2^ ± 0.6×10^-3^ | 0.43 ± 0.34 | 1.11± 0.58 | 17.28 ± 13.51 |

**Table S7** The parameters of detected antibiotics for ecological risk assessment

| Antibiotic | log K_ow_ | algae | | | | |  | invertebrate | | | | |  | fish | | | | |
| --- | --- | --- | --- | --- | --- | --- | --- | --- | --- | --- | --- | --- | --- | --- | --- | --- | --- | --- |
|  |  | Sensitive species | EC_50_ (mg/L) | Types of toxicity | AF | Source |  | Sensitive species | EC_50_ (mg/L) | Types of toxicity | AF | Source |  | Sensitive species | EC_50_ (mg/L) | Types of toxicity | AF | Source |
| NOR | -1.03 | *Microcystis aeruginosa* | 0.062 | chronic (6d) | 100 | ECOTOX |  | Water Flea | 2.39 | chronic (21d) | 100 | Lu et al., 2013 |  | Pimephales promelas | >1000 |  |  | Valcárcel et al., 2011 |
| SA | -0.96 | *Chlorella* | 1.54 | acute (1d) | 1000 | Bialk et al., 2011 |  | / | / | / | / | / |  | / | / | / | / | / |
| OFX | -0.39 | *Microcystis aeruginosa* | 0.021 | chronic (5d) | 100 | ECOTOX |  | Water Flea | 3.13 | chronic (2d) | 100 | Isidori et al., 2005 |  | Danio rerio | >1000 | chronic (2d) |  | Williams et al.,1992 |
| LOM | -0.30 | *Microcystis aeruginosa* | 0.186 | acute (5d) | 100 | Robinson et al., 2004 |  | / | / | / | / | / |  | / | / | / | / | / |
| SDZ | -0.09 | *Microcystis aeruginosa* | 0.135 | chronic (4d) | 100 | ECOTOX |  | Water Flea | 13.7 | chronic (21d) | 100 | Wollenberger et al., 2005 |  | / | / | / | / | / |
| SMZ | 0.14 | *Chlorella* | 11.9 | acute (1d) | 1000 | ECOTOX |  | Water Flea | 277 | chronic (2d) |  | De Liguoro et al., 2009 |  | Oryzias latipes | >100 |  |  | Kim et al., 2007 |
| CIP | 0.28 | *Microcystis aeruginosa* | 0.017 | chronic (5d) | 100 | April et al., 2005 |  | Water Flea | 1.2 | chronic (2d) | 100 | ECOTOX |  | / | / | / | / | / |
| SMT | 0.54 | *Chlorella* | 24.94 | acute (1d) | 1000 | ECOTOX |  | / | / | / | / | / |  | / | / | / | / | / |
| ERX | 0.58 | *Microcystis aeruginosa* | 0.049 | chronic (5d) | 100 | ECOTOX |  | Water Flea | 11.47 | chronic (21d) | 100 | Park et al., 2008 |  | O. latipes | 100 |  |  | Park et al., 2008 |
| SMD | 1.63 | *Chlorella* | 2.3 | chronic (3d) | 100 | Eguchi et al., 2004 |  | / | / | / | / | / |  | / | / | / | / | / |
| SMM | 0.70 | *Isochrysis galbana* | 9.7 | chronic (3d) | 100 | Huang et al., 2014 |  | Kinetoplastid | 100 | acute (1d) | 1000 | Park et al., 2014 |  | / | / | / | / | / |
| SMX | 0.89 | *Microcystis aeruginosa* | 0.55 | acute (1d) | 1000 | ECOTOX |  | Water Flea | 0.21 | chronic (2d) | 100 | Isidori et al., 2005 |  | Rainbow Trout | 27.35 |  |  | Laville et al., 2004 |
| TRI | 0.91 | *Microcystis aeruginosa* | 6.9 | acute (1d) | 1000 | ECOTOX |  | Water Flea | 8.21 | chronic (21d) | 100 | De Liguoro et al., 2012 |  | *Oryzias latipes* | >100 |  |  | Kim et al., 2007 |
| SOX | 1.01 | *Chlorella* | 18.98 | acute (1d) | 1000 | ECOTOX |  | / | / | / | / | / |  | / | / | / | / | / |
| SDM | 1.63 | *Microcystis aeruginosa* | 500 | chronic (6d) | 100 | ECOTOX |  | / | / | / | / | / |  | / | / | / | / | / |
| CLI | 2.16 | *Selenastrum capricornutum* | 0.01 | chronic (3d) | 100 | Ramírez et al., 2022 |  | / | / | / | / | / |  | / | / | / | / | / |
| AZM | 3.03 | *Pseudokirchneriella subcapitata* | 0.019 | chronic (4d) | 100 | Harada et al., 2008 |  | Water Flea | 148 | acute (1d) | 1000 | Li et al., 2020 |  | / | / | / | / | / |
| ROX | 1.70 | *Chlorella* | 3.54 | chronic (7d) | 100 | ECOTOX |  | / | / | / | / | / |  | / | / | / | / | / |
| SPI | 2.99 | *Microcystis aeruginosa* | 0.005 | chronic (7d) | 100 | ECOTOX |  | / | / | / | / | / |  | / | / | / | / | / |
| CLR | 3.16 | *Microcystis aeruginosa* | 0.0046 | chronic (3d) | 100 | Zhang et al., 2023 |  | Water Flea | 25.72 | acute (1d) | 1000 | Isidori et al., 2005 |  | / | / | / | / | / |
| TLS | 1.46 | *Microcystis aeruginosa* | 0.034 | chronic (7d) | 100 | ECOTOX |  | / | / | / | / | / |  | / | / | / | / | / |
| ERY | 2.60 | *Microcystis aeruginosa* | 0.023 | chronic (6d) | 100 | Ando et al., 2007 |  | Water Flea | 210.57 | chronic (2d) | 100 | di Delupis et al.,1992 |  | O. latipes | 100 |  |  | Kim et al., 2007 |
| SMP | 0.35 | *Chlorella* | 5.28 | acute (1d) | 1000 | ECOTOX |  | / | / | / | / | / |  | / | / | / | / | / |
| OXY | -0.90 | *Microcystis aeruginosa* | 0.207 | chronic (7d) | 100 | ECOTOX |  | Water Flea | 46.2 | chronic (21d | 100 | Wollenberger et al., 2000 |  | / | / | / | / | / |
| CTE | -0.13 | *Microcystis aeruginosa* | 73 | chronic (7d) | 100 | ECOTOX |  | / | / | / | / | / |  | / | / | / | / | / |
| TC | -1.30 | *Microcystis aeruginosa* | 0.09 | chronic (7d) | 100 | ECOTOX |  | Water Flea | 111.2 | chronic (2d) | 100 | Kim et al., 2010 |  | Zebra Danio | 127.6 | chronic (3d) |  | Oliveira et al.,2013 |
| DOX | 0.63 | *Tetraselmis chuii* | 22 | chronic (3d) | 100 | Prata et al., 2018 |  | / | / | / | / | / |  | / | / | / | / | / |
| PEN G | 1.83 | *Microcystis aeruginosa* | 0.006 | chronic (7d) | 100 | Halling., 2000 |  | Water Flea | >1000 | chronic (3d) | 100 | Muller et al., 1982 |  | / | / | / | / | / |


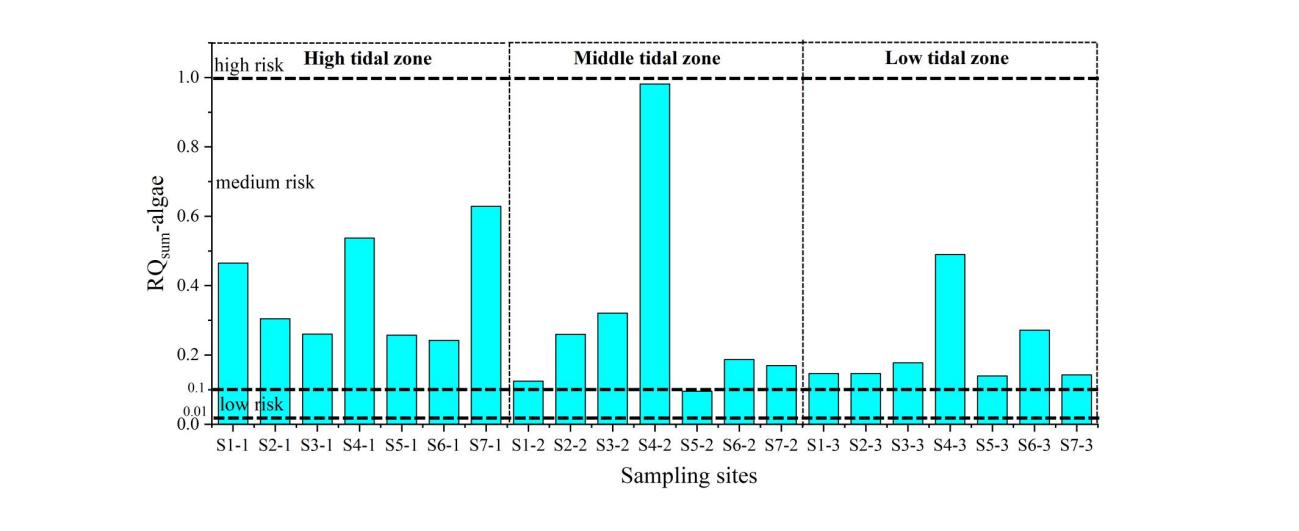


**Fig.S1** Estimated RQ_sum_-algae values of target antibiotics in mangrove sediments of Lianzhou Bay.


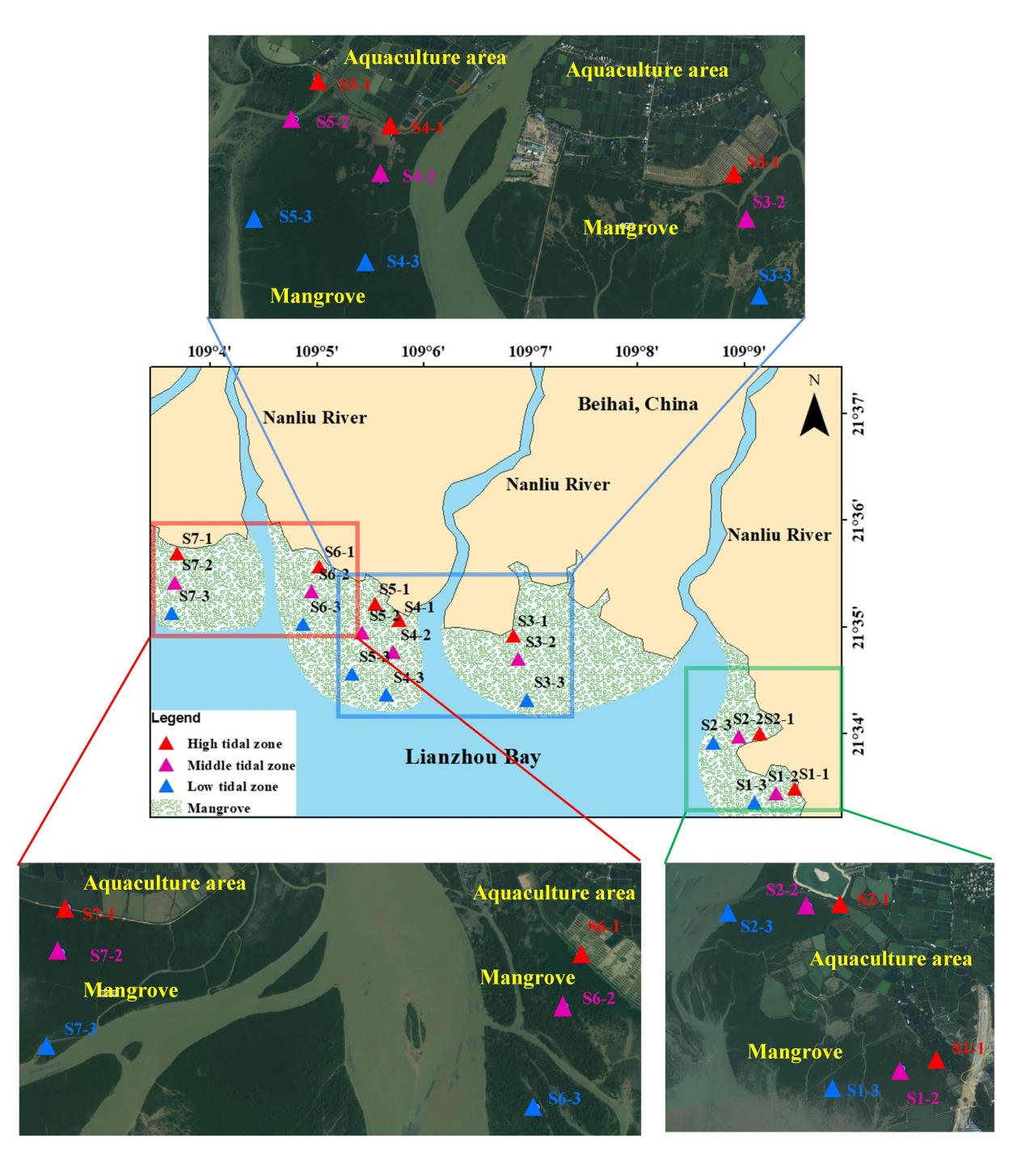


**Fig.S2** Location of sampling sites and the aquaculture area in the mangrove sediments of Lianzhou Bay, China.

**References:**

April, A.R., Jason, B.B., Michael, J.L., 2005. Toxicity of fluoroquinolone antibiotics to aquatic organisms. Environ. Toxicol. Chem. 24, 423-430. <https://doi.org/10.1897/04-210R.1>.

Ando, T., Nagase, H., Eguchi, K., Hirooka, T., Nakamura, T., Miyamoto, K., Hirata, K., 2007. A novel method using cyanobacteria for ecotoxicity test of veterinary antimicrobial agents. Environ. Toxicol. Chem. 26, 601-606. <https://doi.org/10.1897/06-195R.1>.

Brain, R.A., Johnson, D.J., Richards, S.M., Sanderson, H., Sibley, P.K., Solomon, K.R., 2004. Effects of 25 pharmaceutical compounds to Lemna gibba using a seven‐day static‐renewal test. Environ. Toxicol. Chem. 23, 371-382. <https://doi.org/10.1897/02-576>.

Białk-Bielińska, A., Stolte, S., Arning, J., Uebers, U., Böschen, A., Stepnowski, P., Matzke, M., 2011. Ecotoxicity evaluation of selected sulfonamides. Chemosphere, 85, 928-933. [https://doi.org/10.1016/j.chemosphere.2011.06.058](https://doi.org/10.1016/j.chemosphere.2011.06.058).

di Delupis, G.D., Macrí, A., Civitareale, C., Migliore, L., 1992. Antibiotics of zootechnical use: effects of acute high and low dose contamination on Daphnia magna Straus. Aquat. Toxicol. 22, 53-59. [https://doi.org/10.1016/0166-445x(92)90035-l](https://doi.org/10.1016/0166-445x(92)90035-l).

De Liguoro, M., Fioretto, B., Poltronieri, C., Gallina, G., 2009. The toxicity of sulfamethazine to Daphnia magna and its additivity to other veterinary sulfonamides and trimethoprim. Chemosphere, 75, 1519-1524. [https://doi.org/10.1016/j.chemosphere.2009.02.002](https://doi.org/10.1016/j.chemosphere.2009.02.002).

De Liguoro, M., Di Leva, V., Dalla Bona, M., Merlanti, R., Caporale, G., Radaelli, G., 2012. Sublethal effects of trimethoprim on four freshwater organisms. Ecotoxicol. Environ. Safe. 82, 114-121. [https://doi.org/10.1016/j.ecoenv.2012.05.016](https://doi.org/10.1016/j.ecoenv.2012.05.016).

Eguchi, K., Nagase, H., Ozawa, M., Endoh, Y.S., Goto, K., Hirata, K., Miyamoto, K., Yoshimura, H., 2004. Evaluation of antimicrobial agents for veterinary use in the ecotoxicity test using microalgae. Chemosphere. 57, 1733-1738. [https://doi.org/10.1016/j.chemosphere.2004.07.017](https://doi.org/10.1016/j.chemosphere.2004.07.017).

Eguchi, K., Nagase, H., Ozawa, M., Endoh, Y.S., Goto, K., Hirata, K., Miyamoto, K., Yoshimura, H., 2004. Evaluation of antimicrobial agents for veterinary use in the ecotoxicity test using microalgae. Chemosphere. 57, 1733-1738. [https://doi.org/10.1016/j.chemosphere.2004.07.017](https://doi.org/10.1016/j.chemosphere.2004.07.017).

Halling-Sørensen, B., 2000. Algal toxicity of antibacterial agents used in intensive farming. Chemosphere. 40, 731-739. [https://doi.org/10.1016/S0045-6535(99)00445-2](https://doi.org/10.1016/S0045-6535(99)00445-2).

Harada, A., Komori, K., Nakada, N., Kitamura, K., Suzuki, Y., 2008. Biological effects of PPCPs on aquatic lives and evaluation of river waters affected by different wastewater treatment levels. Water. Sci. Technol. 58, 1541-1546. [https://doi.org/10.2166/wst.2008.742](https://doi.org/10.2166/wst.2008.742).

Huang, D., Hou, J., Kuo, T., Lai, H., 2014. Toxicity of the veterinary sulfonamide antibiotic sulfamonomethoxine to five aquatic organisms. Environ. Toxicol. Phar. 38, 874-880. [https://doi.org/10.1016/j.etap.2014.09.006](https://doi.org/10.1016/j.etap.2014.09.006).

Isidori, M., Lavorgna, M., Nardelli, A., Pascarella, L., Parrella, A., 2005. Toxic and genotoxic evaluation of six antibiotics on non-target organisms. Sci. Total. Environ. 346, 87-98. [https://doi.org/10.1016/j.scitotenv.2004.11.017](https://doi.org/10.1016/j.scitotenv.2004.11.017).

Kim, Y., Choi, K., Jung, J.Y., Park, S., Kim, P.G., Park, J., 2007. Aquatic toxicity of acetaminophen, carbamazepine, cimetidine, diltiazem and six major sulfonamides, and their potential ecological risks in Korea. Environ. Int. 33, 370-375. [https://doi.org/10.1016/j.envint.2006.11.017](https://doi.org/10.1016/j.envint.2006.11.017).

Kim, J., Park, J., Kim, P.G., Lee, C., Choi, K., Choi, K., 2010. Implication of global environmental changes on chemical toxicity-effect of water temperature, pH, and ultraviolet B irradiation on acute toxicity of several pharmaceuticals in Daphnia magna. Ecotoxicology, 19, 662-669. [https://doi.org/10.1007/s10646-009-0440-0.](https://doi.org/10.1007/s10646-009-0440-0)

Laville, N., Aıt-Aıssa, S., Gomez, E., Casellas, C., Porcher, J.M., 2004. Effects of human pharmaceuticals on cytotoxicity, EROD activity and ROS production in fish hepatocytes. Toxicology. 196(1-2), pp.41-55. [https://doi.org/10.1016/j.tox.2003.11.002](https://doi.org/10.1016/j.tox.2003.11.002).

Lu, G., Li, Z., Liu, J., 2013. Effects of selected pharmaceuticals on growth, reproduction and feeding of Daphnia Magna. Fresen. Environ. Bull. 22, 2588-2594. [https://doi.org/10.1127/archiv-hydrobiol/116/1989/415.](https://doi.org/10.1127/archiv-hydrobiol/116/1989/415)

Li, Y., Ma, Y., Yang, L., Duan, S., Zhou, F., Chen, J., Liu, Y., Zhang, B., 2020. Effects of azithromycin on feeding behavior and nutrition accumulation of Daphnia magna under the different exposure pathways. Ecotox. Environ. Safe. 197, 110573. [https://doi.org/10.1016/j.ecoenv.2020.110573](https://doi.org/10.1016/j.ecoenv.2020.110573).

Müller, H.G., 1982. Sensitivity of Daphnia magna Straus against eight chemotherapeutic agents and two dyes. Bull. Environ. Contam. Toxicol. 28, 1-2. [https://doi.org/10.1007/bf01608403.](https://doi.org/10.1007/bf01608403)

Oliveira, R., McDonough, S., Ladewig, J.C., Soares, A.M., Nogueira, A.J., Domingues, I., 2013. Effects of oxytetracycline and amoxicillin on development and biomarkers activities of zebrafish (Danio rerio). Environ. Toxicol. Pharmacol. 36, 903-912. [https://doi.org/10.1016/j.etap.2013.07.019](https://doi.org/10.1016/j.etap.2013.07.019).

Park, S., Choi, K., 2008. Hazard assessment of commonly used agricultural antibiotics on aquatic ecosystems. Ecotoxicology. 17, 526-538. [https://doi.org/10.1007/s10646-008-0209-x.](https://doi.org/10.1007/s10646-008-0209-x)

Park, K.H., Zeon, S.R., Lee, J.G., Choi, S.H., Shin, Y.K., Park, K.I., 2014. In vitro and in vivo efficacy of drugs against the protozoan parasite A zumiobodo hoyamushi that causes soft tunic syndrome in the edible ascidian H alocynthia roretzi (Drasche). J. Fish. Dis. 37, 309-317.  <https://doi.org/10.1111/jfd.12104>.

Prata, J.C., Lavorante, B.R., Maria da Conceição, B.S.M., Guilhermino, L., 2018. Influence of microplastics on the toxicity of the pharmaceuticals procainamide and doxycycline on the marine microalgae *Tetraselmis chuii*. Aquat. Toxicol. 197, 143-152. [https://doi.org/10.1016/j.aquatox.2018.02.015](https://doi.org/10.1016/j.aquatox.2018.02.015).

Robinson, A.A., J.B, Belden., M.J, Lydy., 2005. Toxicity of Fluoroquinolone Antibiotics to Aquatic Organisms. Environ. Toxicol. Chem. 24, 423-430.  <https://doi.org/10.1897/04-210R.1>.

Ramírez-Morales, D., Fajardo-Romero, D., Rodríguez-Rodríguez, C.E., Cedergreen, N., 2022. Single and mixture toxicity of selected pharmaceuticals to the aquatic macrophyte Lemna minor. Ecotoxicology. 31, 714-724. [https://doi.org/10.1007/s10646-022-02537-3.](https://doi.org/10.1007/s10646-022-02537-3)

Valcárcel, Y., González Alonso, S., Rodríguez-Gil, J.L., Gil, A., Catalá, M., 2011. Detection of pharmaceutically active compounds in the rivers and tap water of the Madrid Region (Spain) and potential ecotoxicological risk. Chemosphere 84, 1336-1348. [https://doi.org/10.1016/j.chemosphere.2011.05.014](https://doi.org/10.1016/j.chemosphere.2011.05.014).

Williams, R.R., Bell, T.A., Lightner, D.V., 1992. Shrimp antimicrobial testing. II. Toxicity testing and safety determination for twelve antimicrobials with penaeid shrimp larvae. J. Aquat. Anim. Health. 4, 262-270. [https://doi.org/10.1577/1548-8667(1992)004<0262:satitt>2.3.co;2.](https://doi.org/10.1577/1548-8667(1992)004%3c0262:satitt%3e2.3.co;2)

Wollenberger, L., B. Halling-Sorensen., K.O. Kusk, 2000. Acute and Chronic Toxicity of Veterinary Antibiotics to Daphnia magna. Chemosphere. 40, 723-730. [https://doi.org/10.1016/S0045-6535(99)00443-9](https://doi.org/10.1016/S0045-6535(99)00443-9).

Wollenberger, L., Halling-Sørensen, B., Kusk, K.O., 2000. Acute and chronic toxicity of veterinary antibiotics to Daphnia magna. Chemosphere. 40, 723-730. [https://doi.org/10.1016/S0045-6535(99)00443-9](https://doi.org/10.1016/S0045-6535(99)00443-9).

Zhang, Q., Demeestere, K., De Schamphelaere, K.A., 2023. The influence of pH and dissolved organic carbon on the ecotoxicity of ampicillin and clarithromycin. Sci. Tot. Environ. 904, 166781. [https://doi.org/10.1016/j.scitotenv.2023.166781](https://doi.org/10.1016/j.scitotenv.2023.166781).
